# Supplementary material for: Carbon-sink potential of continuous alfalfa agriculture lowered by short-term nitrous oxide emission events
Source: Nat Commun. 2023 Apr 6;14:1926. doi: 10.1038/s41467-023-37391-2 (PMC10079834; doi:10.1038/s41467-023-37391-2)
Supplement: Supplementary file 1 — Supplementary Information [file 41467_2023_37391_MOESM1_ESM.pdf]

Supplementary Materials for

Carbon-sink potential of continuous alfalfa agriculture lowered by short-term nitrous oxide emission events

Authors

Tyler L. Anthony[1]*, Daphne J. Szutu[1], Joseph G. Verfaillie[1], Dennis D. Baldocchi[1], Whendee L. Silver[1]

Affiliations

[1]Ecosystem Science Division, Department of Environmental Science, Policy and Management, University of California at Berkeley, 130 Mulford Hall, Berkeley, CA 94720, USA

***Corresponding author, Email: t.anthony@berkeley.edu**

This PDF file includes:

Supplementary Text
Figs. S1 to S8

Supplemental Results

Diel trends in CH_4 and CO_2 fluxes

Diel trends in soil CH_4 fluxes were variable and not significantly associated with any measured soil characteristic (Fig. 3d). The largest negative CH_4 fluxes and largest variability observed occurred between 1600-2100 hours during the fall; the largest sinks were observed between 900-1200 hours during other seasons. Spring and winter CH_4 fluxes were significantly less negative than in summer and fall (Fig. 3d, $p < 0.001$). Mean annual soil CO_2 fluxes were 4.9 ± 0.01 kg CO_2 m^{-2} y^{-1} and exhibited significant interannual variability with the lowest rates observed in year 4 (Table 1, $p < 0.001$). Soil CO_2 fluxes exhibited similar seasonal and diel trends to mean air temperature (Figs. 3a, 3b, and 5), with emissions significantly higher during the summer and the lowest daily CO_2 fluxes observed in the winter (Fig. 3b, $p < 0.001$). We observed no significant effects of chamber location (plant or bare soil) on overall CO_2, CH_4, or N_2O emissions.

Drivers of soil greenhouse gas emissions

Daily mean N_2O emissions increased by an order of magnitude during extended suboxic periods in February and March 2019 (Fig. 2d). Increases in N_2O emissions also occurred following significant increases in soil moisture at depth (Fig. 2a and 2c), with increases in moisture driven by seasonal rainfall inputs or irrigation events. Short-term (< 24 hour) irrigation events did not always increase soil moisture at depths of 10 cm or greater (Fig. 2d), suggesting increases in N_2O flux following irrigation events were from increased N_2O production in surface soils (< 10 cm).

Methane fluxes exhibited significant wavelet coherence with soil moisture at 50 cm on a daily and weekly scale, and soil moisture at 10 cm at weekly and monthly timescales (Fig. S4, $p < 0.05$). Wavelet coherence also suggested that O_2 concentrations at 10 cm were significantly associated with CH_4 fluxes on daily timescales and O_2 at 50 cm on monthly and seasonal timescales (Fig. S4, $p < 0.05$). Soil temperature had significant coherence with CH_4 fluxes on daily, weekly, monthly, and seasonal timescales across depths, except 10 cm on a daily timescale (Fig. S4, $p < 0.05$). Interactions with soil O_2, and temperature suggested that changes in CH_4 fluxes were generally out of phase with a lagged response across scales.

Daily scale CH_4 fluxes were positively associated with soil moisture and soil O_2 at 10 cm depth and were associated with soil moisture and temperature at 30 and 50 cm depths (Fig. S5, $p < 0.05$). CO_2 fluxes were predominantly associated with soil temperature across all depths at weekly and longer timescales (Fig. S5, $p < 0.05$). Significant positive coherence with soil O_2 was only observed at monthly timescales across depths and seasonal scales at 10 cm (Fig. S5, $p < 0.05$). Soil moisture at 10 cm was significantly positively associated with CO_2 fluxes at weekly timescales and at 10 and 30 cm on seasonal timescales (Fig. S4, $p < 0.05$).

Annual rainfall averaged 330.8 ± 63.5 and ranged from a max of 444 mm y^{-1} in site year 3 to a minimum of 176 mm y^{-1} in site year 4 (Table 3, $p < 0.01$). Annual soil moisture values (0-50 cm) were highest in year 2, significantly decreasing in years 3 and 4 (Table 3, 1$p < 0.001$). Soil NO_3^-

concentrations averaged 4.9 ± 1.3 µg NO_3^- g $soil^{-1}$ and weekly variation in soil NO_3^- values was not associated with increased N_2O emissions (Fig. 2b). Weekly soil NH_4^+ concentrations averaged 11.5 ± 0.8 µg NH_4^+ g $soil^{-1}$ and were also not significantly associated with soil fluxes (Fig. 4b). Weekly soil pH values from April 2018-April 2019 averaged 5.82 ± 0.01, with weekly means ranging from 5.26 ± 0.03 in early October 2018 to 6.31 ± 0.04 in March 2019.

Plant phenology and greenhouse gas fluxes

We observed significant differences in NIRv following alfalfa cutting events (Fig. 5c) and annual mean NIRv decreased significantly across the measurement period (Table 3, $p < 0.001$). Wavelet coherence analysis suggested that CO_2 fluxes were only significantly associated with NIRv as a lagged response at biweekly and seasonal scales (Fig. S3a, $p < 0.05$). Similar relationships were observed between NIRv and soil CH_4 fluxes, but also included significant coherence at the biannual scale (Fig. S3b, $p < 0.05$). Only N_2O was significantly positively associated with NIRv at daily and seasonal scales (Fig. S3c, $p < 0.05$).

Depth trends in soil greenhouse gas concentrations

Daily mean soil N_2O fluxes were positively correlated with soil atmospheric N_2O concentrations across soil depths (Fig. S1, 10 cm $R^2 = 0.60$, $p < 0.001$, 30 cm $R^2 = 0.53$, $p < 0.001$, 50 cm $R^2 = 0.45$, $p < 0.001$). No significant relationships were observed between soil CO_2 and CH_4 concentrations and net CO_2 and CH_4 fluxes (Figs. S6 and S7).

Measurements of soil gas concentrations indicated significant temporal variability in soil CO_2 concentrations and high N_2O and CH_4 concentration events within the soil environment (Fig. S8). The peak in soil atmosphere N_2O concentrations corresponded to the onset of soil inundation following substantial rainfall, which led to a significant increase in soil moisture and significant decrease in soil O_2 across soil depths (Fig. 2c and 2d). The observed peak in soil CH_4 concentrations occurred near the end of this soil saturation event. The peaks in soil N_2O and CH_4 did not correspond to significant hot moments of net soil N_2O and CH_4 emissions. Measurements of soil atmospheric CO_2 concentrations were generally high (> 10,000 ppm) throughout the year, particularly at depth (30 and 50 cm). Soil CO_2 concentration decreased significantly following rainfall events in March-April 2019. Rainfall during this period generated extended soil waterlogging that reduced soil O_2 concentrations (Fig. 4d). However, soil CO_2 concentrations exhibited no statistical relationships with soil CO_2 fluxes (Fig. S6).

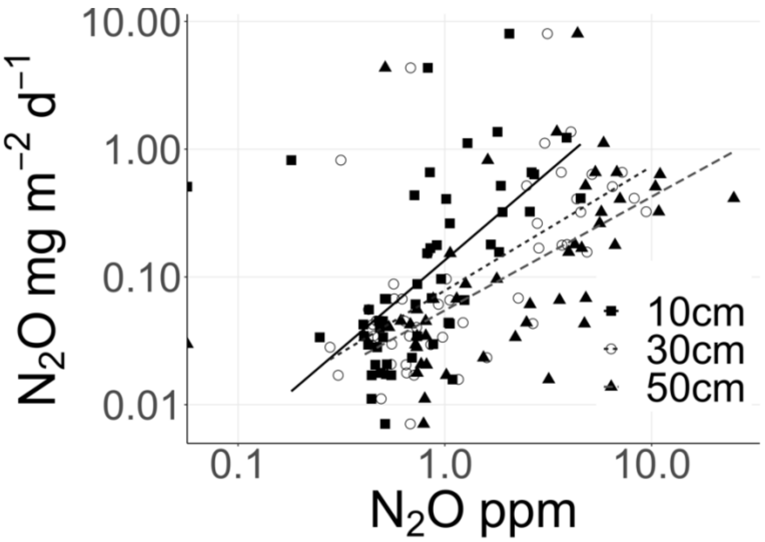

Fig. S1. Linear regressions of daily mean soil nitrous oxide (N_2O) fluxes (mg m^{-2} d^{-1}, n = approximately 80 per day) and soil atmosphere N_2O concentrations (parts per million, ppm) across soil depths (10 cm: squares, 30 cm: open circles, and 50 cm: triangles) with daily mean soil N_2O flux in mg m^{-2} d^{-1} (10 cm: $R^2 = 0.60$, $F(1,24) = 52.9$, $p < 0.001$, 30 cm: $R^2 = 0.53$, $F(1,22) = 100.8$, $p < 0.001$, 50 cm: $R^2 = 0.45$, $F(1,22) = 425.3$, $p < 0.001$).

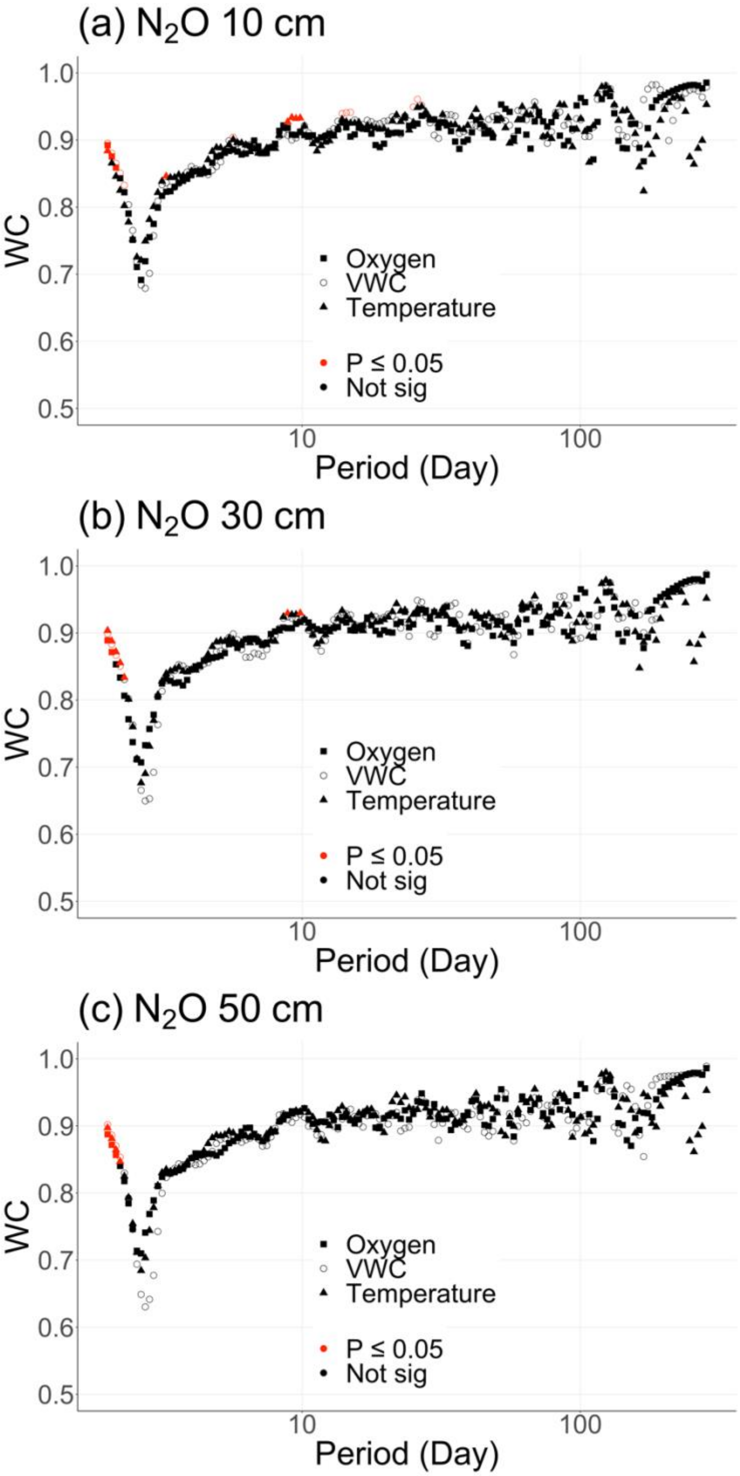

Fig. S2. Wavelet coherence (WC) for soil nitrous oxide (N_2O) fluxes (mg N_2O m^{-2} d^{-1}) with O_2 (%) concentrations (squares), soil temperature (°C, triangles), and daily mean soil moisture (%, open circles) at 10 cm (a), 30 cm (b) and 50 cm (c). Each point represents the average wavelet coherence across a range of periodicities (period = day). Red highlights indicate significant coherence ($p < 0.05$). Significance is determined with a > 95% confidence interval of 1000 Monte Carlo simulations.

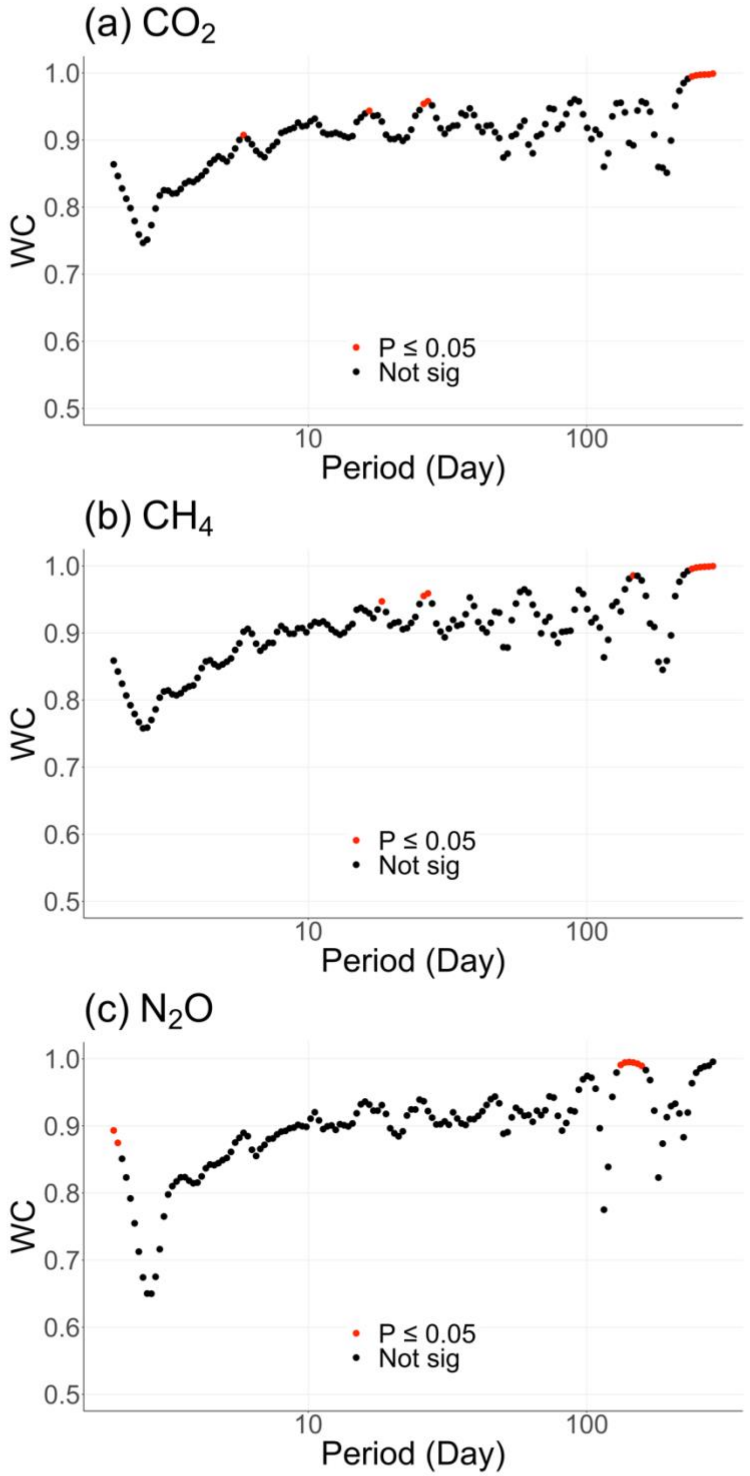

Fig. S3. Wavelet coherence (WC) for (a) soil nitrous oxide (N_2O, mg N_2O m^{-2} d^{-1}), (b) methane (CH_4, mg CH_4 m^{-2} d^{-1}) and (c) carbon dioxide (CO_2, g CO_2 m^{-2} d^{-1} fluxes with daily mean near-infrared reflectance of vegetation (NIRv). Each point represents the average wavelet coherence across a range of periodicities (period = day). Red highlights indicate significant coherence ($p < 0.05$). Significance is determined with a > 95% confidence interval of 1000 Monte Carlo simulations.

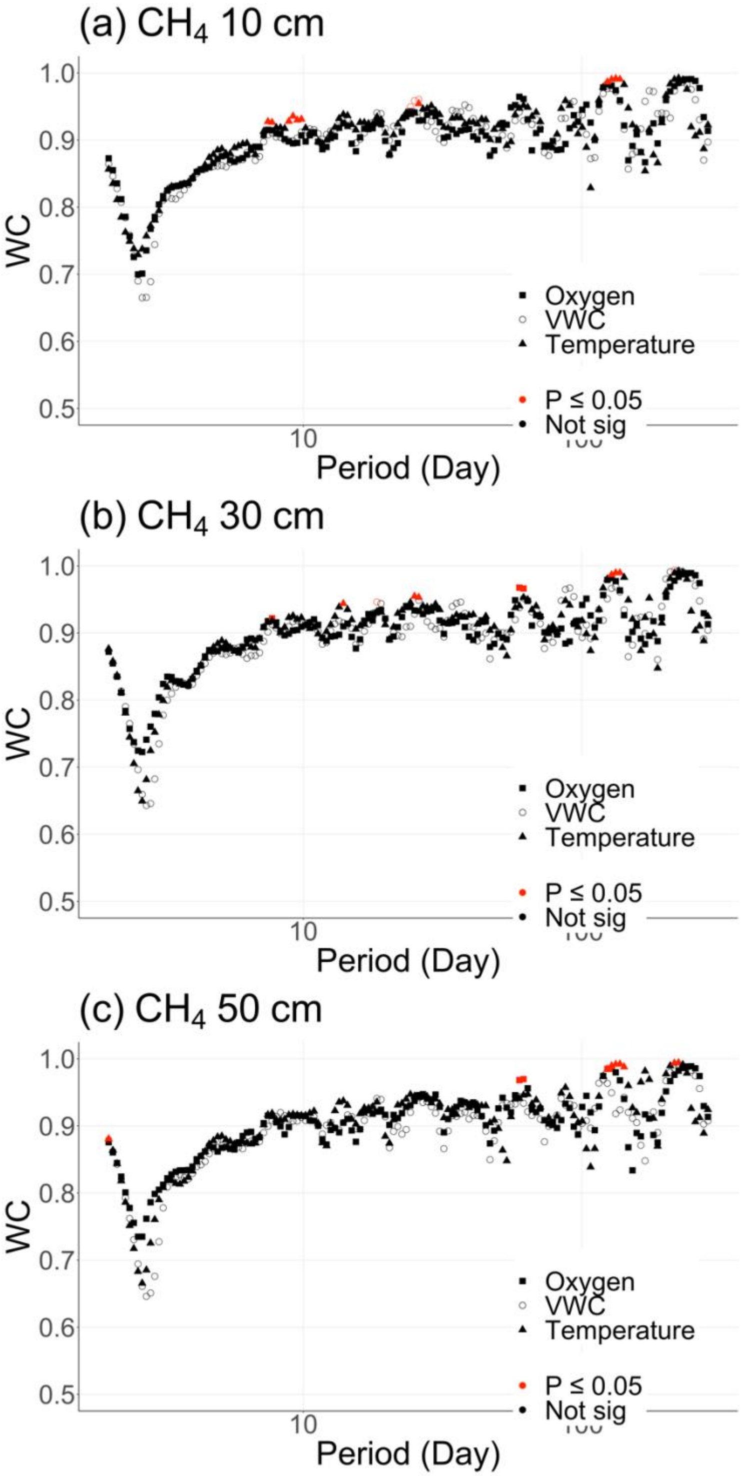

Figure S4. Wavelet coherence (WC) for soil methane (CH_4, mg CH_4 m^{-2} d^{-1}), fluxes with daily mean oxygen (O_2 (%), squares), soil temperature (°C, triangles), and soil moisture (%, open circles) at 10 cm (a), 30 cm (b) and 50 cm (c). Each point represents the average wavelet coherence across a range of periodicities (period = day). Red highlights indicate significant coherence ($p < 0.05$). Significance is determined with a > 95% confidence interval of 1000 Monte Carlo simulations.

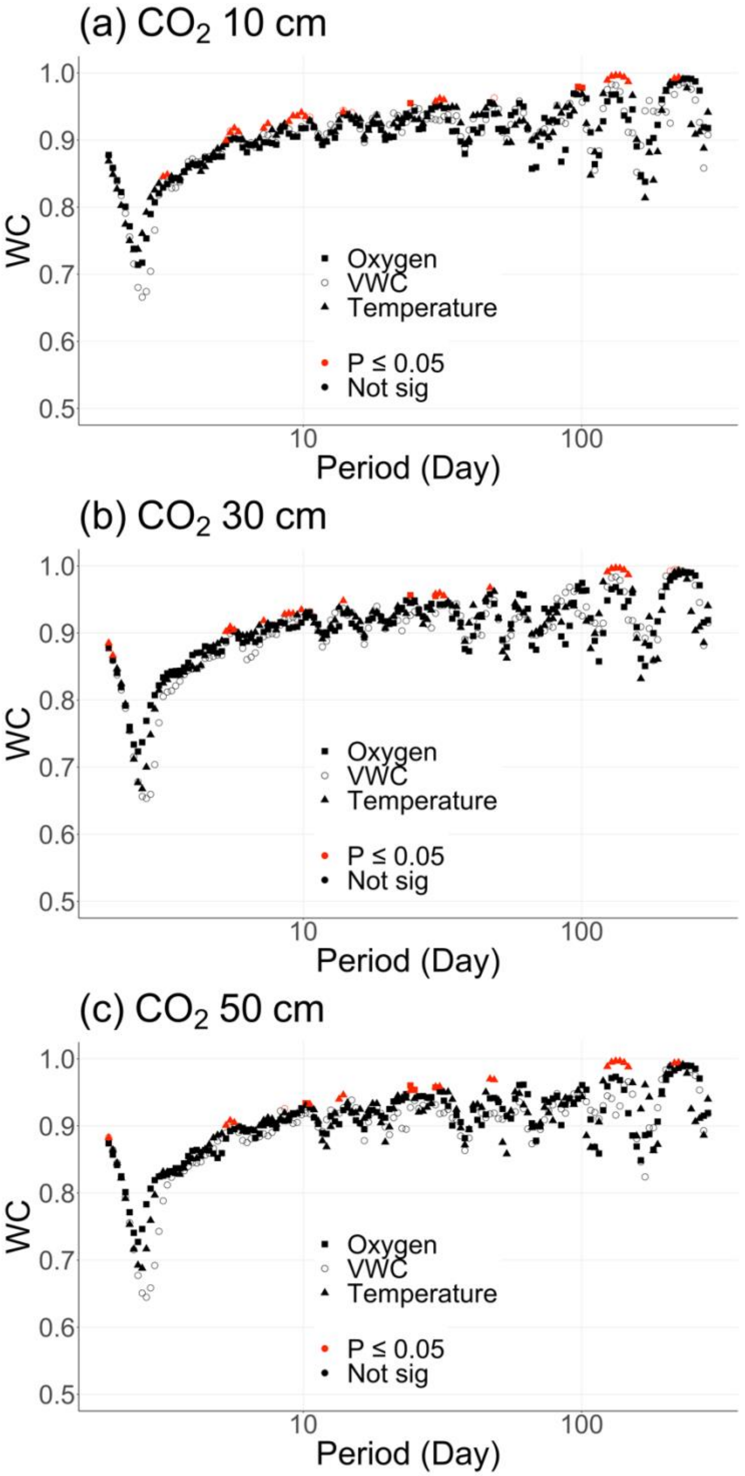

Figure S5. Wavelet coherence (WC) for soil carbon dioxide (CO_2) fluxes with daily mean oxygen (O_2 (%) squares), soil temperature (°C, triangles), and soil moisture (%, open circles) at 10 cm (a), 30 cm (b) and 50 cm (c). Each point represents the average wavelet coherence across a range of periodicities (period = day). Red highlights indicate significant coherence ($p < 0.05$). Significance is determined with a > 95% confidence interval of 1000 Monte Carlo simulations.

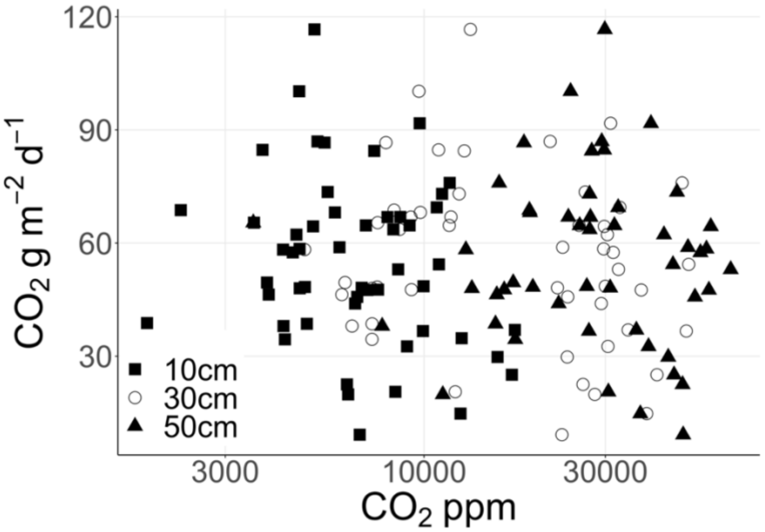

Figure S6. Relationships between daily mean soil carbon dioxide (CO_2) flux (g m^{-2} d^{-1}) and soil atmosphere CO_2 concentrations (parts per million, ppm) across soil depths (10 cm: squares, 30 cm: open circles, and 50 cm: triangles).

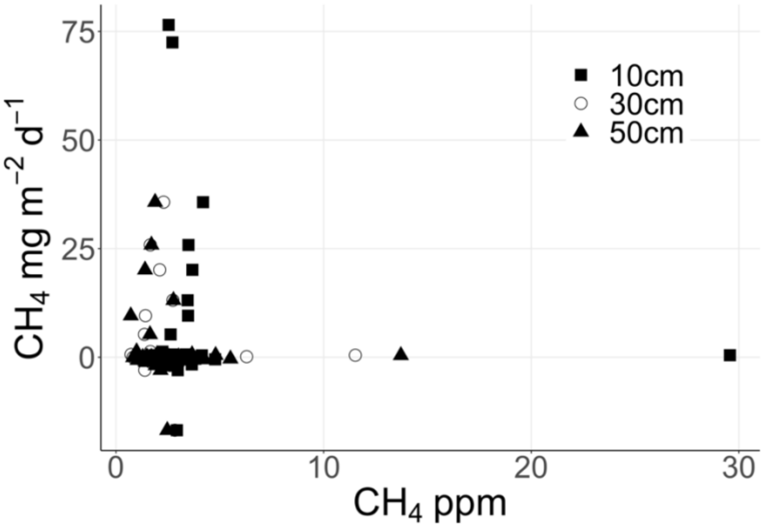

Figure S7. Relationships between daily mean soil methane (CH_4) flux (mg m^{-2} d^{-1}) and soil atmosphere CH_4 concentrations (parts per million, ppm) across soil depths (10 cm: squares, 30 cm: open circles, and 50 cm: triangles).

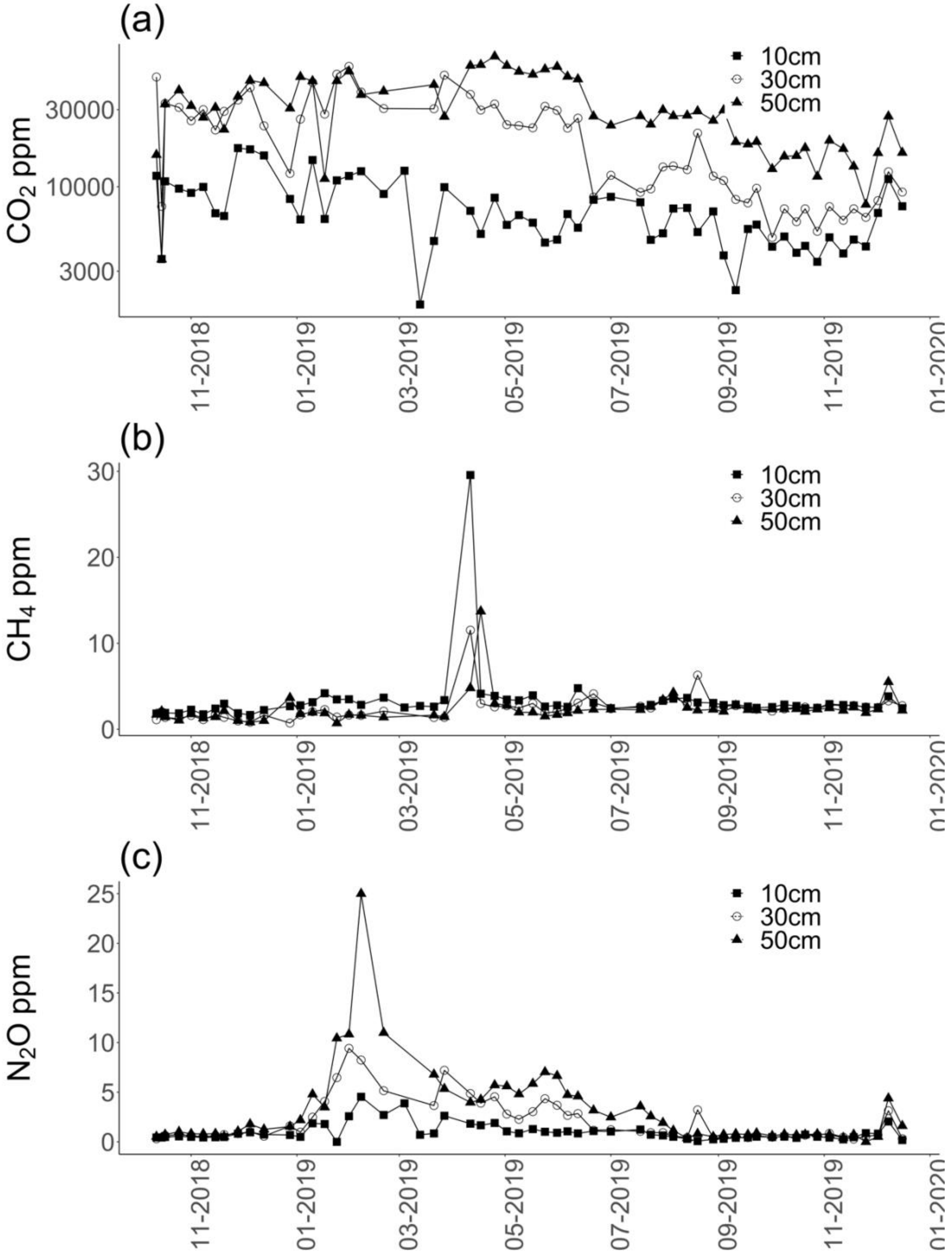

Figure S8. Daily mean (± SE) (a) carbon dioxide (CO_2 parts per million, ppm) concentrations, (b) methane (CH_4 parts per million, ppm)) concentrations, and (c) nitrous oxide (N_2O parts per million, ppm)) concentrations across soil depths. Depth values are labeled as squares (10 cm), open circles (30 cm), and triangles (50 cm).
