# Supplementary material for: Carbon-sink potential of continuous alfalfa agriculture lowered by short-term nitrous oxide emission events
Source: Nat Commun. 2023 Apr 6;14:1926. doi: 10.1038/s41467-023-37391-2 (PMC10079834; doi:10.1038/s41467-023-37391-2)
Supplement: Supplementary file 2 — Reporting Summary [file 41467_2023_37391_MOESM2_ESM.pdf]

nature portfolio

Corresponding author(s): Tyler L. Anthony

Last updated by author(s): Mar 13, 2023

Reporting Summary

Nature Portfolio wishes to improve the reproducibility of the work that we publish. This form provides structure for consistency and transparency in reporting. For further information on Nature Portfolio policies, see our Editorial Policies and the Editorial Policy Checklist.

Statistics

For all statistical analyses, confirm that the following items are present in the figure legend, table legend, main text, or Methods section.

n/a	Confirmed	
☐	☒	The exact sample size (*n*) for each experimental group/condition, given as a discrete number and unit of measurement
☐	☒	A statement on whether measurements were taken from distinct samples or whether the same sample was measured repeatedly
☐	☒	The statistical test(s) used AND whether they are one- or two-sided<br>*Only common tests should be described solely by name; describe more complex techniques in the Methods section.*
☐	☒	A description of all covariates tested
☐	☒	A description of any assumptions or corrections, such as tests of normality and adjustment for multiple comparisons
☐	☒	A full description of the statistical parameters including central tendency (e.g. means) or other basic estimates (e.g. regression coefficient) AND variation (e.g. standard deviation) or associated estimates of uncertainty (e.g. confidence intervals)
☐	☒	For null hypothesis testing, the test statistic (e.g. F, t, r) with confidence intervals, effect sizes, degrees of freedom and P value noted<br>*Give P values as exact values whenever suitable.*
☒	☐	For Bayesian analysis, information on the choice of priors and Markov chain Monte Carlo settings
☐	☒	For hierarchical and complex designs, identification of the appropriate level for tests and full reporting of outcomes
☐	☒	Estimates of effect sizes (e.g. Cohen's *d*, Pearson's *r*), indicating how they were calculated

Our web collection on statistics for biologists contains articles on many of the points above.

Software and code

Policy information about availability of computer code

Data collection	Flux calculations and analyses were first performed using Eosense eosAnalyze-AC v. 3.7.7 software, then data quality assessment and control were subsequently performed in R (RStudio, v.1.1.4633).
Data analysis	Following data filtering, all statistical analyses were performed using JMP Pro 15 (SAS Institute Inc., Cary, NC). Differences in site year, hourly, and seasonal mean flux values were analyzed with one-way ANOVAs followed by post-hoc Tukey tests. Values reported in the text are means ± standard errors unless otherwise noted.<br>For wavelet coherence, missing flux data were replaced with zeroes to compute an unbiased estimator of the wavelet variance for time series with missing observations. Statistical significance (p-value) was computed using 1000 Monte Carlo simulations. All wavelet decomposition and coherence calculations were conducted using the WaveletComp 1.1 R package. All data filtering code has been deposited in the link under our Code Availability statement.

For manuscripts utilizing custom algorithms or software that are central to the research but not yet described in published literature, software must be made available to editors and reviewers. We strongly encourage code deposition in a community repository (e.g. GitHub). See the Nature Portfolio guidelines for submitting code & software for further information.

Data

Policy information about availability of data

All manuscripts must include a data availability statement. This statement should provide the following information, where applicable:

- Accession codes, unique identifiers, or web links for publicly available datasets
- A description of any restrictions on data availability
- For clinical datasets or third party data, please ensure that the statement adheres to our policy

All greenhouse gas flux and soil sensor data are available as Source Data deposited in a Dryad dataset: https://datadryad.org/stash/share/igfrCACBTOMTNEi8KsL3auVLnSqKiN51WFRUlFf04Ds

Human research participants

Policy information about studies involving human research participants and Sex and Gender in Research.

Reporting on sex and gender	N/A
Population characteristics	N/A
Recruitment	N/A
Ethics oversight	N/A

Note that full information on the approval of the study protocol must also be provided in the manuscript.

Field-specific reporting

Please select the one below that is the best fit for your research. If you are not sure, read the appropriate sections before making your selection.

☐ Life sciences ☐ Behavioural & social sciences ☒ Ecological, evolutionary & environmental sciences

For a reference copy of the document with all sections, see nature.com/documents/nr-reporting-summary-flat.pdf

Ecological, evolutionary & environmental sciences study design

All studies must disclose on these points even when the disclosure is negative.

Study description	Continuous surface fluxes of N2O, CH4, and CO2 were measured continuously from January 2017 to February 2021 using an automated chamber system. The system consisted of nine opaque, automated gas flux chambers (eosAC, Eosense, Nova Scotia, Canada) connected to a multiplexer (eosMX, Eosense, Nova Scotia, Canada). The multiplexer allowed for dynamically signaled chamber deployment and routed gases to a cavity ring-down spectrometer (Picarro G2508, Santa Clara, CA, USA). Chambers (n = 9) represented individual sampling replicates within the field to account for spatial heterogeneity. Chambers were randomly assigned to either plant rows (n = 5) or inter-plant areas of bare soil (n = 4), estimated to each represent ~50% of the ecosystem's soil surface. Chambers were measured sequentially over a 10-min sampling period with a 1.5-min flushing period before and after each measurement. All individual chamber data were nested within the alfalfa ecosystem flux dataset.
Research sample	Individual automated chamber flux measurements from an irrigated alfalfa field. Chambers were deployed in a 10 x 10 m grid design, with each chamber approximately 5 m from other chambers and as far from the edge of the field as possible (20-30 m) to limit edge effects. Extended 15 cm soil collars were utilized to maintain measurement collection and ensure chambers were not inundated during irrigation or high rainfall events. Chambers were randomly assigned to either plant rows (n = 5) or inter-plant areas of bare soil (n = 4). Chambers remained in their original positions throughout the field campaign, except for short periods (< 3 days) during field management activities (e.g., harvest, winter grazing).
Sampling strategy	Sampling strategy was to maximize the number of individual flux measurements to create an accurate representation of annual greenhouse gas budgets. Sample size was determined by instrumentation performance and field activities, which limited the number of samples collected during each annual monitoring period. These sample sizes are sufficient because they represent the longest continuously monitored fluxes from any continuous alfalfa ecosystem, and the sampling strategy was optimized to collect as many samples as instrument limitations allowed. Foliage near chambers were minimally trimmed as needed between harvests if it inhibited chamber closure.To determine chamber volume, chamber collar heights were measured approximately weekly and interpolated between measurements to account for changes in chamber height over time.
Data collection	Data was collected from Eosense eosMX software installed on a cavity ring-down spectrometer (Picarro G2508, Santa Clara, CA, USA) which also recored raw greenhouse gas concentrations. TLA collected and processed both soil sensor, recorded on a Campbell Scientific CR1000 (Campbell Scientific, Logan, UT, USA) datalogger, and greenhouse gas data data approximately weekly. TLA then recorded processed data into corresponding data files.

Timing and spatial scale	Start dates: January 26th 2017, end date: January 26, 2021. During instrument operation, fluxes were captured approximately every 15 minutes. The spatial extent of the campaign was a 10 m x 10 m grid, with each chamber sampling a 182 cm^2 area.
Data exclusions	Fluxes were removed with erroneous spectrometer cavity temperature or pressure values or negative concentrations, corresponding to instrument malfunction. Fluxes were removed if deployment period was < 9 or > 11 min, indicative of chamber malfunction. Linear and exponential fluxes were compared using estimate uncertainty to estimate ratios. Data filtering removed 2.1% of flux measurement periods, generating a final dataset of 108,638, 103,013, and 102,997 simultaneous flux measurements of CO2, N2O, and CH4 respectively
Reproducibility	Samples were collected continuously (individual chambers sampled approximately once every 2 hours) from approximately the same location throughout the monitoring period. However, due to inherent climate and land management variability it is impossible to recreate the results exactly. Chambers were randomly assigned to either plant rows (n = 5) or inter-plant areas of bare soil (n = 4) and placed sufficiently far apart (~5 m) to ensure sampling was not influenced by the presence of other chambers.
Randomization	Chambers were randomly assigned to either plant rows (n = 5) or inter-plant areas of bare soil (n = 4). Chambers remained in their original positions throughout the field campaign, except for short periods (< 3 days) during field management activities (e.g., harvest, winter grazing)
Blinding	Blinding is not relevant or possible in an environmental monitoring study as its not appropriate for the research questions asked here.

Did the study involve field work? ☒ Yes ☐ No

Field work, collection and transport

Field conditions	These individual continuous field measurements for 4 complete years (January 2017-January 2021). The site had a Mediterranean climate with hot dry summers and cool wet winters. The region's historical mean annual temperature was 15.1 ± 6.3 °C and a mean annual rainfall averaging 326 ± 4 mm. Site year (January 27 - January 26) rainfall data was collected from a nearby (< 1 km) Ameriflux site.
Location	The study was conducted in continuous alfalfa in Bouldin Island in the Sacramento-San Joaquin Delta region of California, USA (38.11ºN, 121.5ºW).
Access & import/export	Site access was provide by the California Department of Water Resources and the Metropolitan Water District of Southern California, accessed via a maintained dirt access road in a university owned vehicle . Samples were collected by hand within the alfalfa field and were transported back to the lab in a university owned vehicle within one hour of collection.
Disturbance	Harvesting events were minimally disrupted by chambers on the field, this was quickly mediated by increased site monitoring after the first event.

Reporting for specific materials, systems and methods

We require information from authors about some types of materials, experimental systems and methods used in many studies. Here, indicate whether each material, system or method listed is relevant to your study. If you are not sure if a list item applies to your research, read the appropriate section before selecting a response.

Materials & experimental systems

n/a	Involved in the study	
☒	☐	Antibodies
☒	☐	Eukaryotic cell lines
☒	☐	Palaeontology and archaeology
☒	☐	Animals and other organisms
☒	☐	Clinical data
☒	☐	Dual use research of concern

Methods

n/a	Involved in the study	
☒	☐	ChIP-seq
☒	☐	Flow cytometry
☒	☐	MRI-based neuroimaging
